# How to dose extended-release carbidopa-levodopa capsules (IPX203, CREXONT®) in patients with Parkinson’s disease

**DOI:** 10.1016/j.prdoa.2025.100357

**Published:** 2025-06-18

**Authors:** Robert A. Hauser, Yasar Torres-Yaghi, Stanley Fisher, Ghazal Banisadr

**Affiliations:** a University of South Florida, Tampa, FL, United States; b MedStar Georgetown University Hospital, Washington, DC, United States; c Amneal Pharmaceuticals, LLC, 400 Crossing Boulevard, Bridgewater, NJ, United States

**Keywords:** CREXONT, IPX203, Levodopa, Extended-Release, Parkinson’s Disease, Treatment, Dosing

## Abstract

IPX203 (CREXONT®) is a novel, oral, extended-release (ER) carbidopa-levodopa (CD-LD) formulation, designed to rapidly achieve LD plasma concentrations similar to immediate-release (IR) CD-LD and to maintain LD concentrations for a longer duration than other oral CD-LD products. In the pivotal phase 3 clinical trial, IPX203 provided superior clinical benefit with fewer doses than IR CD-LD. Since the plasma concentration profile of IPX203 is different from that of IR LD, converting patients treated with IR LD to IPX203 requires a conversion strategy. In the pivotal phase 3 trial, patients on IR CD-LD were converted to IPX203 based on the most frequent single dose of the patient’s stable dosing regimen of IR CD-LD. IPX203 was initially administered 3 times per day for most patients. Further titration was then undertaken based on clinical response, and patients were dosed 2 to 4 times per day. Of 589 patients previously treated with IR CD-LD, 506 patients completed the conversion to IPX203; the mg conversion ratio for individual doses was 2.8 at the beginning of the dose-conversion period and 2.9 after titration to clinical response, with the majority of IPX203 patients (84%) dosed 3 times per day.

**Objective:**

To provide guidance on dosing of IPX203 (CREXONT®) in clinical practice.

## Highlights

• IPX203 is designed to address LD’s short half-life and limited area for absorption.
• IPX203 dosages aren’t interchangeable 1:1 with other LD formulations.
• Clinicians must familiarize themselves with IPX203 dosing and administration.
• We provide essential guidance on the dosing of IPX203 in clinical practice.

## 1 Introduction

IPX203 is a novel, oral, extended-release (ER) formulation of carbidopa (CD) and levodopa (LD), developed to address the short half-life and limited area for absorption of LD in the gastrointestinal tract. IPX203 contains immediate-release (IR) granules and ER pellets that provide rapid absorption of LD to achieve the desired plasma concentration and maintain LD plasma levels above 50 % C_max_ for longer than can be achieved with other oral LD formulations [[1], [2], [3]]. IPX203 was designed to prolong LD absorption in the gut by including a mucoadhesive polymer to allow pellets to adhere to the mucosa in the proximal small intestine area for a longer time.

It is critically important to recognize that dosages of IPX203 are not interchangeable on a 1:1 mg basis with IR CD-LD. Therefore, those wishing to prescribe IPX203 must familiarize themselves with its proper dosing and administration.

IPX203 is the investigational name; the brand name in the United States is CREXONT®.

## 2 Dosage strengths available

**Fig. 1:**
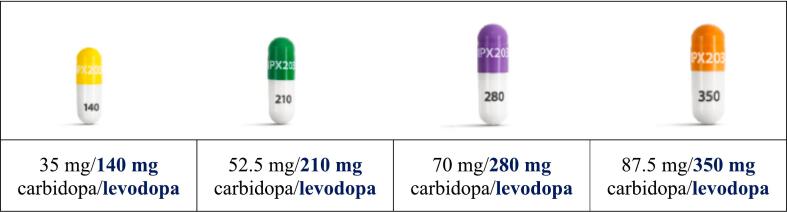
IPX203 dose strengths. Capsules not shown to scale.

IPX203 contains CD and LD in a 1:4 ratio. Available dosage strengths are ER capsules (Fig. 1):

• 35 mg CD and 140 mg LD: Yellow and white capsule imprinted with IPX203 on the capsule cap and 140 on the capsule body; capsule size 2
• 52.5 mg CD and 210 mg LD: Green and white capsule imprinted with IPX203 on the capsule cap and 210 on the capsule body; capsule size 1el
• 70 mg CD and 280 mg LD: Purple and white capsule imprinted with IPX203 on the capsule cap and 280 on the capsule body; capsule size 0el
• 87.5 mg CD and 350 mg LD: Medium orange and white capsule imprinted with IPX203 on the capsule cap and 350 on the capsule body; capsule size 00

For dosing purposes, only the LD dosage usually needs to be considered and that is what will be discussed here unless otherwise indicated. The LD 140 mg, 210 mg, 280 mg, and 350 mg dosage capsules were developed to help avoid confusion with other oral CD-LD products.

## 3 Dosing conversion for patients on immediate-release carbidopa-levodopa

In the phase 3 clinical trial [4], patients on IR CD-LD (Sinemet) were converted to IPX203 according to the dosage conversion table provided (Table 1).

**Table 1:** Conversion table from IR CD-LD to IPX203 used in the phase 3 clinical trial [3,5].

**Most frequent IR CD-LD** **single dose (mg of LD)**	**Recommended starting IPX203 single dose (mg of LD)**	**Recommended starting IPX203 dose frequency**^a^
100 mg	280 mg	three times daily
150 mg	420 mg	three times daily
200 mg	560 mg	three times daily
>200 mg	700 mg	three times daily

Abbreviation: IR CD-LD, immediate-release carbidopa-levodopa; LD, levodopa.

a Patients on a total daily dose of less than 125–500  mg IR CD-LD at the end of the dose-adjustment period were advised to initially take IPX203 twice daily. After starting treatment with IPX203, the dosage (mg) and dosing frequency could be increased to three times daily if the subject did not achieve an acceptable duration of effect.

The first step was to identify the most frequent single dose of IR CD-LD (mg of LD) and convert to IPX203 (mg of LD) by multiplying by 2.8, for single doses up to 250 mg. If the most frequent single dose was > 250 mg IR LD, subjects were initiated on 700 mg, but the dose could be increased if required. In the pivotal trial, when two or more IR CD-LD doses corresponded to the most frequent, the suggested IPX203 conversion was based on the higher of the two IR CD-LD doses [4]. The recommended starting frequency for IPX203 was three times daily (TID) unless the patient was taking < 500 mg IR LD per day. Patients on a total daily IR LD dose < 500 mg were advised to initially take IPX203 twice daily (BID). After starting treatment with IPX203, the individual dosage strength (mg) and dosing frequency were adjusted further as clinically warranted, up to a maximum dosing frequency of four times daily (QID).

Importantly, to simplify study drug manufacturing requirements, the pivotal phase 3 study included only 140 mg IPX203 capsules. In clinical practice, where all four dosage strengths of IPX203 are available, the following initial conversion table can be used to minimize patient pill burden throughout the day (Table 2).

**Table 2:** Conversion table from IR CD-LD to IPX203 that can be used in clinical practice.

1 Identify the starting IPX203 dose based on most frequent single IR CD-LD dose			

**Most frequent** **IR CD-LD single dose**	**Suggested starting** **IPX203 single dose**	**Conversion to IPX203**	

25/100 mg	70/280 mg	1x	

37.5/150 mg	105/420 mg	2x	

50/200 mg	140/560 mg	2x	

>50/200 mg	175/700 mg	2x	

2 Initiate patients on the above individual IPX203 dose TID. For patients receiving a total daily dose of < 500 mg IR LD, initiate IPX203 BID.			

3 Adjust the dosing regimen to achieve optimal balance of efficacy and tolerability by minimizing “Off” time without causing troublesome dyskinesia or other dopaminergic side effects. IPX203 can be dosed two to four times daily based on patient response.			

Abbreviation: BID, twice daily; IR CD-LD, immediate-release carbidopa-levodopa; LD, levodopa; TID, three times daily.

By way of example, if a patient is taking five IR CD-LD doses per day, three of which are IR CD-LD 25/100 (1 tab) and two of which are IR CD-LD 37.5/150 (1 and ½ tabs), the most frequent individual dose of IR LD would be 100 mg and the suggested initial conversion would be IPX203 70/280 taken TID. After the initial dosing, further adjustments would then be made based on the clinical response.

It is very important to tell patients that the initial conversion is a suggested starting point, and further adjustments might be required. Initial follow-up is recommended 1 to 3 days following conversion to IPX203 and multiple adjustments can be made until an optimal dosing regimen is achieved.

In the phase 3 clinical trial, 452 (77 %) of the 589 patients who were converted from IR CD-LD to IPX203 started on TID dosing. Of these, 52 (12 %) increased to QID dosing and the remainder continued TID dosing. Fifty-four (9.2 %) patients were converted from IR CD-LD to IPX203 and started on BID dosing; 31 (57 %) of these patients remained on twice daily IPX203 through the end of the conversion period, 21 (39 %) increased to TID dosing, and 2 (4 %) increased to QID dosing. The average number of titration steps —either a dose and/or frequency adjustment— to stable dosing on IPX203 was 1.6.

**Fig. 2:**
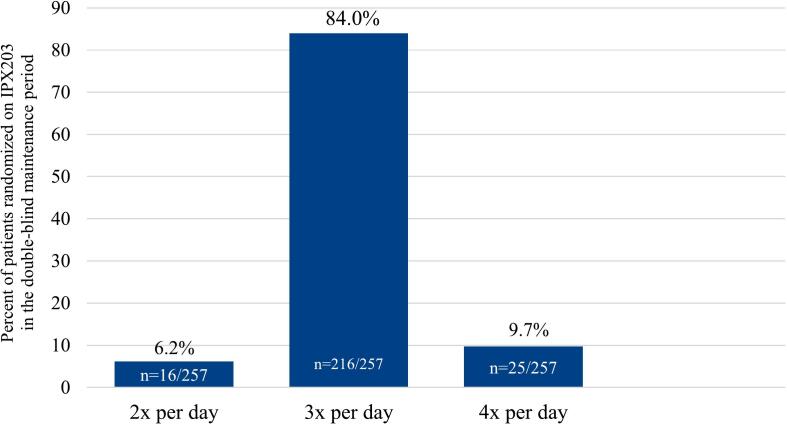
IPX203 distribution of dosing frequency during randomized double-blind period.

The mean (SD) daily dosing frequency of IPX203 at the end of the phase 3 study was 3.04 (0.40) times per day. Sixteen patients (6.2 %) took it BID, 216 (84 %) took it TID, and 25 (9.7 %) took it QID (Fig. 2). The study protocol dictated that the shortest time allowed between doses was 6 h, which limited the number of patients who took it QID. It is anticipated that in clinical practice, some patients will require dosing QID with dosing more frequently than every 6 h to achieve an optimal response throughout the day.

Overall, in the phase 3 trial, the mean ratio of IPX203 LD total daily dose (TDD) to IR LD TDD was 1.79 at end of study (EOS), and the mean ratio of IPX203 most frequent single dose to IR LD most frequent single dose was 2.92 [4]. There are two reasons for this. First, IPX203 has slightly lower bioavailability —ranging from 88 % to 99 % based on area under the curve (AUC)— compared to IR CD-LD [2,3]. Second, more LD is required to fill in the LD pharmacokinetic troughs created by IR CD-LD and to smooth the clinical response. When converting patients with motor fluctuations from IR CD-LD to IPX203, one would typically like to match the serum LD maximum concentration (C_max_) to provide the same level of antiparkinsonian benefit during “On” time, without increasing dyskinesia. Since the C_max_ of IPX203 is approximately 38 % that of IR CD-LD [1,2], it was suggested that a single dose of LD in IPX203 should be 2.8 times the amount of LD in IR formulation. This conversion factor would be expected to provide a IPX203 C_max_ slightly above that provided by the IR CD-LD dose (0.38 x 2.8 = 1.06).

As with titration of other LD products, if a suggested individual IPX203 dose does not provide a good “On” response, that dose should be increased. If a dose doesn’t last until the next dose “kicks in,” the inter-dose interval should be shortened. The dosing frequency of IPX203 can be changed from BID up to QID if more frequent dosing is needed and tolerated. The approved maximum recommended daily dosage of IPX203 is 2100 mg per day [5]. In the phase 3 clinical trial, the most common administration frequency of IPX203 was TID, but this may have been artificially low due to protocol limitations, and it is expected that some of these patients would have achieved more clinical benefit with four doses per day.

Some patients, especially those who are more advanced and more sensitive to changes in LD dosage, will notice a difference within 1 to 3 days after conversion from IR CD-LD. Therefore, clinicians must have a plan in place for patient feedback and be prepared to make adjustments quickly if the patient is underdosed (parkinsonian) or overdosed (dyskinetic).

## 4 Additional dosing considerations

For patients converting to IPX203 from Rytary, it is recommended to initiate IPX203 on an approximate 1:1 mg basis of Rytary, using the LD component [5]. Clinicians should use their clinical judgment to determine the dosing frequency.

For patients on CD-LD and a catechol-O-methyltransferase (COMT) inhibitor (eg, entacapone or opicapone), the initial total daily dose of IPX203 may need to be increased if the COMT inhibitor is discontinued [5].

In the RISE-PD study, many participants were receiving concomitant therapy with other anti-parkinsonian medications, including dopamine agonists, selective monoamine oxidase type B inhibitors, amantadine, and anticholinergic drugs provided the doses were stable for at least 4 weeks prior to screening and throughout the study [4]. The use of IPX203 in combination with other LD products has not been studied [5].

## 5 Hypothetical cases

The four hypothetical cases presented here are based on clinical experience and are presented for illustrative purposes and intended as general guidance. Each individual patient should be adjusted as needed based on clinical judgment.

### 5.1 CASE 1

Patient is a 66-year-old woman who is taking IR CD-LD 25/100 mg five times a day: 1.5 tablets at 6:00 AM, and then 1 tablet at 10:00 AM, 2:00 PM, 6:00 PM, and 10:00 PM. She states that it takes approximately 30 min for each dose to kick in and each dose lasts approximately 3.5 h, leaving an hour of “Off” time between doses and a total of 4.5 h of “Off” time per day. She is converted to IPX203 by identifying the most frequent IR LD dose (100 mg) and multiplying by 2.8 to get the individual IPX203 LD dose, which is 280 mg (100 x 2.8 = 280). Because her total daily IR LD dose was ≥ 500 mg per day, IPX203 CD-LD 70/280 mg is then administered TID. In this case, the physician suggests dosing at 6:00 AM, noon, and 6:00 PM.

The patient calls the physician’s office after 2 days and states that each dose works well and the pills last about 5 h per dose. She notes that each dose takes approximately 30 min to kick in and lasts approximately 5 h, but she still has an hour of “Off” time between doses. She also notes that she develops symptoms if she goes to bed late or if she awakens during the night. Her dosing schedule is then changed to 6:00 AM, 11:00 AM, 4:00 PM, and 9:00 PM. On this schedule, the patient now reports that IPX203 is working well and lasts from dose to dose with no wearing off. She also stays “On” even if she goes to bed late or awakens early in the night.

### 5.2 CASE 2

Patient is a 62-year-old man who is taking IR CD-LD 25/100 mg four times a day, 2 tablets at 7:00 AM, 1.5 tablets at 11:00 AM, 2 tablets at 3:00 PM, and 1.5 tablets at 7:00 PM. He reports that he is experiencing approximately 1 h of “Off” time between doses. In this case, there are two single IR LD doses (150 mg and 200 mg) that are “tied” for most frequent. When two or more IR CD-LD doses correspond to the most frequent, the conversion is based on the higher of the two, which in this case is 200 mg. Since his total daily IR LD dose is ≥ 500 mg per day, he is converted to IPX203 70/280 mg, 2 capsules (560 mg LD) TID at 7:00 AM, noon, and 5:00 PM. He calls the office after two days noting that his new regimen has eliminated “Off” periods between doses, but he is experiencing unwanted “twisting, turning movements” for several hours a day. His physician explains that this IPX203 dose is too high for him and is causing dyskinesia. The physician then provides a new prescription for IPX203 52.5/210 mg, 2 capsules (420 mg LD) TID at 7:00 AM, noon, and 5:00 PM. When seen in follow-up, the patient reports that this dose “kicks in” the morning after approximately 30 min, as his previous IR regimen did, but now lasts from dose to dose with minimal extra movements (dyskinesia) that are not bothersome.

### 5.3 CASE 3

Patient is a 60-year-old woman who is taking IR CD-LD 25/100 mg along with entacapone 200 mg QID at 8:00 AM, 11:30 AM, 3:00 PM, and 6:30 PM. She also takes controlled-release (CR) CD-LD 50/200 mg at bedtime. She reports being “Off” for an hour in between LD doses. Her care provider recommends converting her to IPX203 to replace her IR and CR LD, as well as her entacapone. She is converted to IPX203 by identifying the most frequent IR LD dose (100 mg), multiplying by 1.3 to calculate the LD equivalent dose of IR LD + entacapone [6] (100 x 1.3 = 130), and then multiplying by 2.8 to get the individual IPX203 LD dose, which is 364 mg (130 x 2.8 = 364). As the closest available IPX203 dose strength is 350 mg, the patient was started on 350 mg of IPX203 TID at 7:00 AM, 1:00 PM, and 7:00 PM. On contact from the physician’s office 3 days later, the patient stated that she was still experiencing wearing off. Therefore, her dosing regimen was adjusted to 350 mg IPX203 QID at 7:00 AM, noon, 5:00 PM, and 10:00 PM.

In the phase 3 clinical trial, patients could not enter the trial if they were taking daytime doses of CR CD-LD but could be taking a single bedtime dose of CR CD-LD. In those cases, the CR CD-LD dose was discontinued and initially substituted with a 1:1 mg-equivalent dose of IR CD-LD that was subsequently included in the dosing conversion to IPX203. In clinical practice, a bedtime dose of CR CD-LD does not need to be converted to IR CD-LD before conversion to IPX203 and can be considered in the conversion as if it were IR CD-LD. In this case, the patient was taking a bedtime CR CD-LD dose of 200 mg LD, considered “equivalent” to 200 mg IR CD-LD with regard to the IPX203 conversion. Here, the single bedtime dose of CR CD-LD did not affect the determination of the most frequent IR CD-LD dose as it was the only 200 mg dose and there were four 100 mg IR CD-LD doses.

### 5.4 CASE 4

Patient is a 73-year-old man who is taking 2 capsules of Rytary 195 mg QID (at 7:00 AM, noon, 5:00 PM, and 10:00 PM). He is also taking pramipexole 0.5 mg QID and rasagiline 1 mg qAM. He states that he experiences 30 min of “Off” time between LD doses. He also notes that he experiences an hour of mild dyskinesia with each Rytary dose. To convert patients from Rytary to IPX203, they are switched on an approximately 1:1 mg basis using the LD component for conversion [5]. In this case, the closest IPX203 dose strength to his current individual Rytary dose (2 x 195 LD mg = 390) is 2 capsules of IPX203 210 mg (2 x 210 = 420). He was started on 2 capsules of IPX203 210 mg QID (at 7:00 AM, noon, 5:00 PM, and 10:00 PM). He continued his pramipexole and rasagiline. During follow-up, the patient reported persistent dyskinesia and was switched to one capsule of IPX203 350 mg QID (at 7:00 AM, noon, 5:00 PM, and 10:00 PM). The patient responded well to the new dosing regimen with benefit lasting from dose to dose with minimal dyskinesia. In this case, increasing the individual doses of LD from Rytary 390 mg to IPX203 420 mg caused increased and more prolonged dyskinesia. Lowering the individual doses of IPX203 from 420 mg to 350 mg likely lowered the LD C_max_ and reduced the dyskinesia, while the IPX203 formulation maintained plasma LD levels for a longer time than was achieved with Rytary and allowed reduction in “Off” time.

## 6 Duration of benefit per dose

An analysis of data from the phase 3 clinical trial found that IPX203 provided 1.6 h longer duration of benefit (“Good On” time) *per dose* compared to IR CD-LD [4]. This data may help physicians plan dosing regimens and set expectations when converting from IR CD-LD to IPX203.

## 7 Pill burden

IPX203 provides an opportunity to reduce both dosing frequency and daily pill burden. A retrospective pill burden analysis was performed for the phase 3 trial [4]. To make a double-blind, double-dummy comparison feasible in the phase 3 trial, IR CD-LD tablets were limited to 25–100 mg CD-LD and IPX203 capsules were limited to 35–140 mg CD-LD. Each IPX203 capsule and each IR CD-LD tablet or partial tablet were counted as one pill. Results showed that the mean number of pills taken per day was 10.6 for the IPX203 group and 8.7 for the IR CD-LD group. However, a hypothetical pill burden analysis was also performed that evaluated daily pill burden if the marketed dosage strengths for IPX203 (140, 210, 280, and 350 mg) and the 25–250 mg dosage strength for IR CD-LD would have been available in the clinical trial. Results of this analysis showed that the mean number of pills taken per day would have been 5.4 for the IPX203 group and 7.9 for the IR CD-LD group (Table 3). Minimizing pill burden after the dosing regimen is optimized can be an important step in maintaining patient adherence.

**Table 3:** IPX203 daily pill count.

**Daily pill count in the RISE-PD study, restricted to the IPX203 140 mg pill strength**		
	**IPX203**	**IR CD-LD**
N	256	250
Mean	10.6	8.7
SD	4.22	3.84
Median	10.0	8.0
Min	3	4
Max	24	21

**Expected daily pill count across all four available IPX203 strengths in a real-world clinical setting**		

	**IPX203**	**IR CD-LD**
N	256	250
Mean	5.4	7.9
SD	1.74	2.95
Median	6.0	8.0
Min	2	4
Max	12	20

## 8 Dosing in patients naïve to levodopa therapy

In patients naïve to LD, all LD formulations may be effective, and a long-term advantage of any one of them over another has not been proven. There remains interest in the notion that long-acting LD formulations might forestall and reduce the development of motor complications (fluctuations and dyskinesias) [7] and a need remains for long-term comparative clinical trials. IPX203 is approved for use in LD-naïve patients and potential candidates include those who prefer less frequent dosing and those who need LD therapy but are at high risk or concerned about the development of motor complications. For patients naïve to LD therapy, the recommended initial dosage of IPX203 is 140 mg BID for the first 3 days. Thereafter, if the response is suboptimal, the dosing can be increased gradually as needed to a maximum total daily dose of 2100 mg divided up to 4 times daily.

## 9 Administration information

IPX203 can be taken with or without food. The quickest and most consistent absorption occurs when taken away from food. Protein can reduce absorption, but the clinical relevance depends on how sensitive to small dose changes the patient is.

In healthy adults, oral administration of IPX203 after a high-fat, high-calorie meal increased C_max_ by approximately 19 % and AUC_0-∞_ by approximately 18 % for LD compared to administration in the fasted state. There might be a delay of approximately 2 h in reaching C_max_ when IPX203 is taken with a high-fat, high-calorie meal [3]. The study also demonstrated that sprinkling the capsule contents on applesauce did not alter the pharmacokinetic profile of LD, compared to administration of the intact capsule [3]. However, while this alternative method of administration may support effective dosing in patients who have difficulty swallowing capsules, it is not currently recommended in the FDA-approved labeling. According to the prescribing information, IPX203 should be swallowed whole, without chewing, dividing, or crushing [5].

## 10 Conclusion

Compared to IR CD-LD, IPX203 is approximately 88 % to 99 % bioavailable by AUC and 38 % bioavailable by C_max_ [1,5]. Patients with motor fluctuations on IR CD-LD can be initially converted to IPX203 by multiplying the most frequent single dose of IR CD-LD (up to 250 mg) by 2.8, given TID, followed by titration according to clinical response. It is critical to inform patients that the initial regimen is just a starting dosage, and subsequent adjustments based on clinical response may be needed after 1 to 3 days and may be repeated until an optimal dosing regimen is achieved. Patients naïve to LD can be initiated on IPX203 140 mg BID for the first 3 days; the dosage may then be increased gradually as needed.

## Funding and Conflicts of Interest

This work was supported by Amneal Pharmaceuticals, LLC, Bridgewater, NJ, in accordance with Good Publication Practice Guidelines. The authors did not receive payment for the development of this publication. S.F. and G.B. are employees of Amneal Pharmaceuticals.

## Data Availability Statement

Data from this study will be shared according to regulatory guidelines and timelines (eg, on ClinicalTrials.gov), and as determined by Amneal Pharmaceuticals. De-identified patient data can only be shared by people other than Amneal Pharmaceuticals after written approval from Amneal Pharmaceuticals.

## CRediT authorship contribution statement

**Robert A. Hauser:** Writing – review & editing, Writing – original draft, Investigation, Data curation, Conceptualization. **Yasar Torres-Yaghi:** Writing – review & editing. **Stanley Fisher:** Writing – review & editing, Data curation, Conceptualization. **Ghazal Banisadr:** Writing – review & editing, Writing – original draft, Project administration, Data curation, Conceptualization.

## Declaration of competing interest

The authors declare that they have no known competing financial interests or personal relationships that could have appeared to influence the work reported in this paper.
